# The Training of Midwives

**Published:** 1886-12-04

**Authors:** 


### THE TRAINING OF MIDWIVES.

THE Secretary to the Queen Charlotte Lying-In Hospital writes: "With reference to the twofold question under the above head, in THE HOSPITAL of this date, I beg to say that there is no lying-in hospital, in the metropolis at any rate, where midwifery may be learnt gratis, but a course of theoretical and practical training, twelve weeks in duration, embracing a thorough preparation for the examination of the Obstetrical Society of London, may be undergone at this Institution for a fee of £26 5*s*., including board and lodging. I do not think there is a lying-in hospital in the United Kingdom wealthy enough to provide such a training free of charge. THOMAS RYAN.

---

THE Superintendent of the Belgravia Institute for Trained Nurses, 25, Lupus Street, S.W., writes: "In reply to the query of H. C. H. regarding the cost of training as a midwife, I beg to inform her that there is *no* lying-in hospital in which she could be trained *free*. The smallest fees, I believe, are those at the Brownlow Hill Infirmary, Liverpool, and the Royal Maternity Hospital, Edinburgh, in either of which, I think, the cost of training does not exceed £10. In the London hospitals it varies from £20 to £25 for the course. It seems to me a great pity that the lying-in wards of our London infirmaries cannot be made available for the tuition of midwives, at reduced fees. Perhaps when the long-hoped-for Bill enforcing the Licensing and Registration of Midwives by Government is passed, we may get something of this kind.

ELINOR AGNES BEDINGFELD,
Accoucheuse Diplomée.

---

THE Honorary Secretary of the Workhouse Infirmary Nursing Association, 44, Berners Street, W., writes: "In reply to the question in last week's HOSPITAL, regarding free training in maternity work, I beg to say that the Workhouse Infirmary Nursing Association gives to nurses who are already trained in *general* nursing, midwifery training gratis, on condition that they bind themselves to the Association for the three years following their training. The next vacancy will be in January. Particulars can be obtained from me any Friday morning from 11 to 1 a.m. Candidates are expected to pass the Obstetric examination.

L. WILSON.

